# Supplementary material for: Preclinical and early clinical safety of intra-articular spheroid adipose-derived stem cells for knee osteoarthritis: A translational study
Source: Osteoarthr Cartil Open. 2026 Mar 28;8(2):100792. doi: 10.1016/j.ocarto.2026.100792 (PMC13090708; doi:10.1016/j.ocarto.2026.100792)
Supplement: Multimedia component 1 [file mmc1.pptx]

## Slide 1
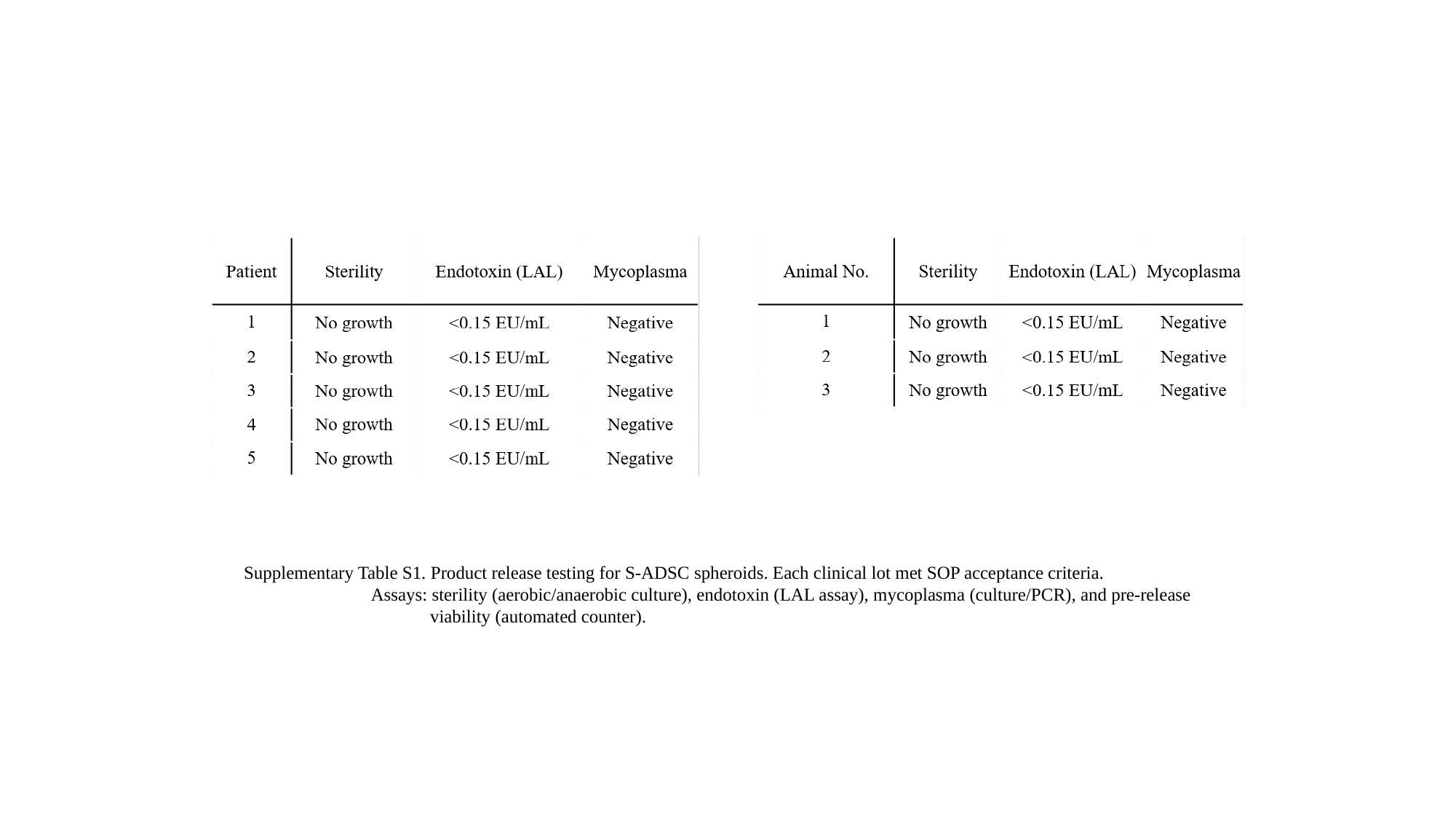

Supplementary Table S1. Product release testing for S‑ADSC spheroids. Each clinical lot met SOP acceptance criteria.
 Assays: sterility (aerobic/anaerobic culture), endotoxin (LAL assay), mycoplasma (culture/PCR), and pre‑release
 viability (automated counter).

## Slide 2
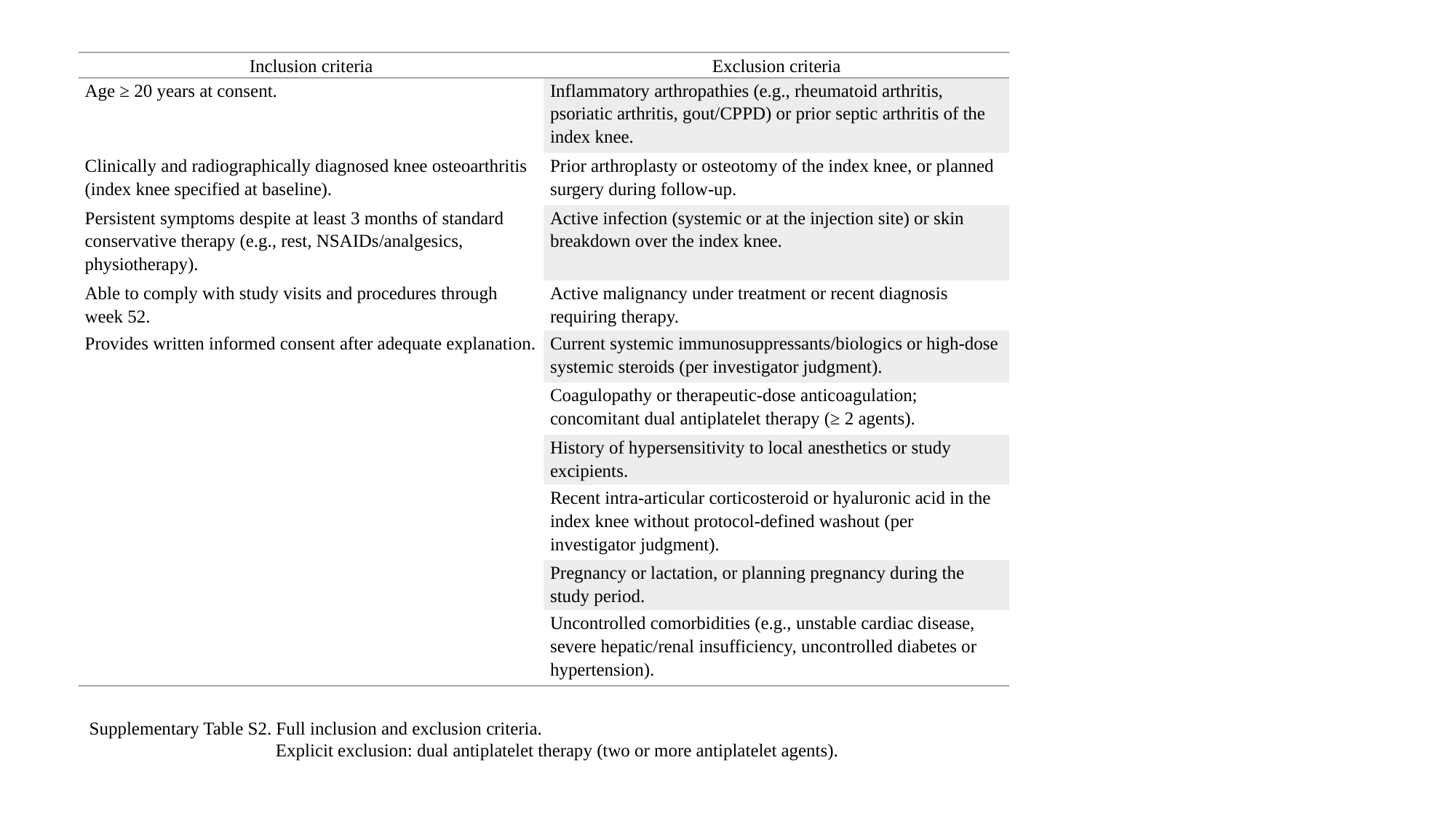

| Inclusion criteria | Exclusion criteria |
| --- | --- |
| Age ≥ 20 years at consent. | Inflammatory arthropathies (e.g., rheumatoid arthritis, psoriatic arthritis, gout/CPPD) or prior septic arthritis of the index knee. |
| Clinically and radiographically diagnosed knee osteoarthritis (index knee specified at baseline). | Prior arthroplasty or osteotomy of the index knee, or planned surgery during follow‑up. |
| Persistent symptoms despite at least 3 months of standard conservative therapy (e.g., rest, NSAIDs/analgesics, physiotherapy). | Active infection (systemic or at the injection site) or skin breakdown over the index knee. |
| Able to comply with study visits and procedures through week 52. | Active malignancy under treatment or recent diagnosis requiring therapy. |
| Provides written informed consent after adequate explanation. | Current systemic immunosuppressants/biologics or high‑dose systemic steroids (per investigator judgment). |
| | Coagulopathy or therapeutic‑dose anticoagulation; concomitant dual antiplatelet therapy (≥ 2 agents). |
| | History of hypersensitivity to local anesthetics or study excipients. |
| | Recent intra‑articular corticosteroid or hyaluronic acid in the index knee without protocol‑defined washout (per investigator judgment). |
| | Pregnancy or lactation, or planning pregnancy during the study period. |
| | Uncontrolled comorbidities (e.g., unstable cardiac disease, severe hepatic/renal insufficiency, uncontrolled diabetes or hypertension). |
Supplementary Table S2. Full inclusion and exclusion criteria.
 Explicit exclusion: dual antiplatelet therapy (two or more antiplatelet agents).

## Slide 3
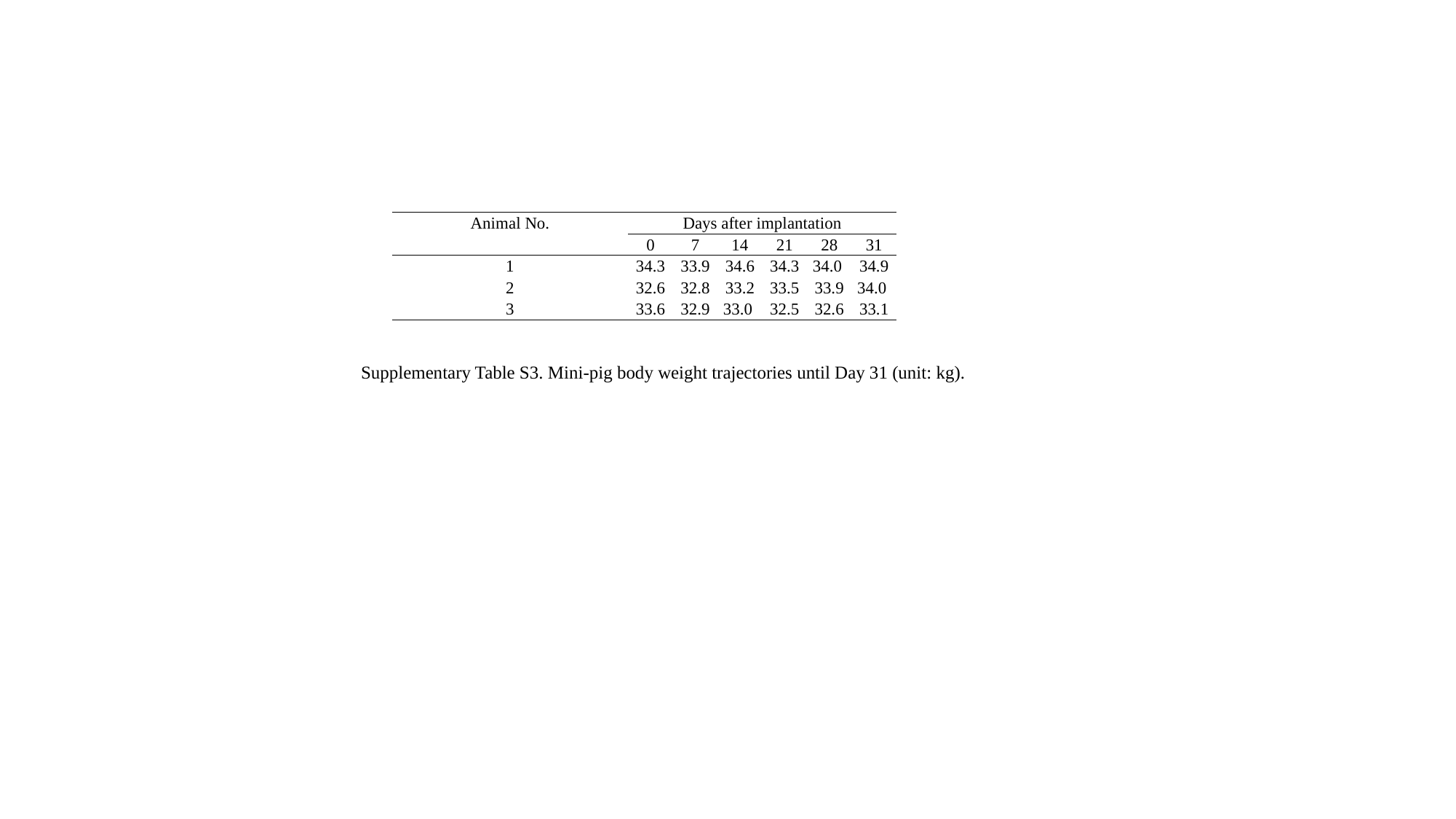

| Animal No. | Days after implantation | | | | | |
| --- | --- | --- | --- | --- | --- | --- |
| | 0 | 7 | 14 | 21 | 28 | 31 |
| 1 | 34.3 | 33.9 | 34.6 | 34.3 | 34.0 | 34.9 |
| 2 | 32.6 | 32.8 | 33.2 | 33.5 | 33.9 | 34.0 |
| 3 | 33.6 | 32.9 | 33.0 | 32.5 | 32.6 | 33.1 |
Supplementary Table S3. Mini‑pig body weight trajectories until Day 31 (unit: kg).

## Slide 4
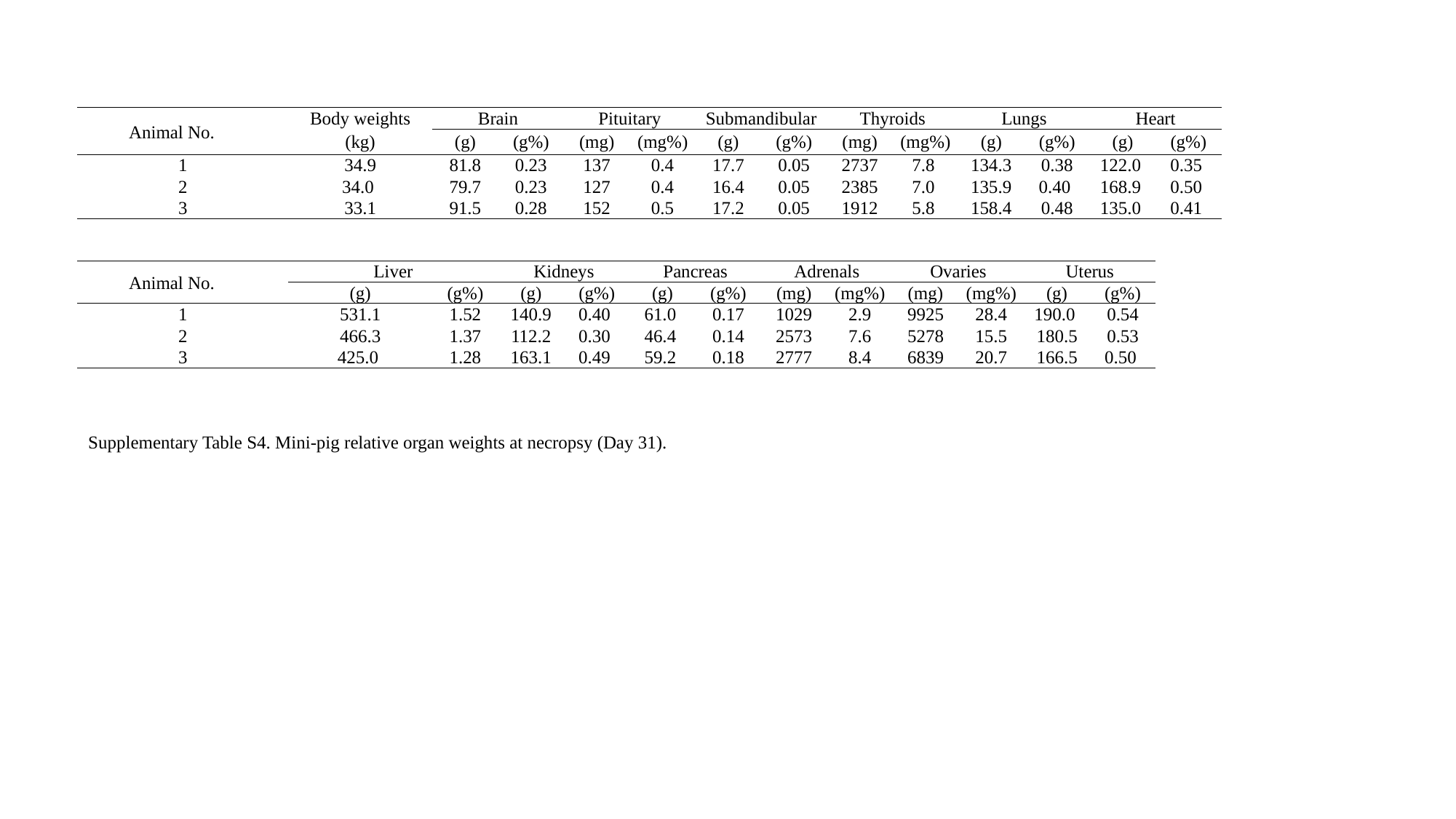

| Animal No. | Body weights | Brain | | Pituitary | | Submandibular | | Thyroids | | Lungs | | Heart | |
| --- | --- | --- | --- | --- | --- | --- | --- | --- | --- | --- | --- | --- | --- |
| | (kg) | (g) | (g%) | (mg) | (mg%) | (g) | (g%) | (mg) | (mg%) | (g) | (g%) | (g) | (g%) |
| 1 | 34.9 | 81.8 | 0.23 | 137 | 0.4 | 17.7 | 0.05 | 2737 | 7.8 | 134.3 | 0.38 | 122.0 | 0.35 |
| 2 | 34.0 | 79.7 | 0.23 | 127 | 0.4 | 16.4 | 0.05 | 2385 | 7.0 | 135.9 | 0.40 | 168.9 | 0.50 |
| 3 | 33.1 | 91.5 | 0.28 | 152 | 0.5 | 17.2 | 0.05 | 1912 | 5.8 | 158.4 | 0.48 | 135.0 | 0.41 |
| | | | | | | | | | | | | | |
| | | | | | | | | | | | | | |
| Animal No. | Liver | | Kidneys | | Pancreas | | Adrenals | | Ovaries | | Uterus | | |
| | (g) | (g%) | (g) | (g%) | (g) | (g%) | (mg) | (mg%) | (mg) | (mg%) | (g) | (g%) | |
| 1 | 531.1 | 1.52 | 140.9 | 0.40 | 61.0 | 0.17 | 1029 | 2.9 | 9925 | 28.4 | 190.0 | 0.54 | |
| 2 | 466.3 | 1.37 | 112.2 | 0.30 | 46.4 | 0.14 | 2573 | 7.6 | 5278 | 15.5 | 180.5 | 0.53 | |
| 3 | 425.0 | 1.28 | 163.1 | 0.49 | 59.2 | 0.18 | 2777 | 8.4 | 6839 | 20.7 | 166.5 | 0.50 | |
Supplementary Table S4. Mini‑pig relative organ weights at necropsy (Day 31).

## Slide 5
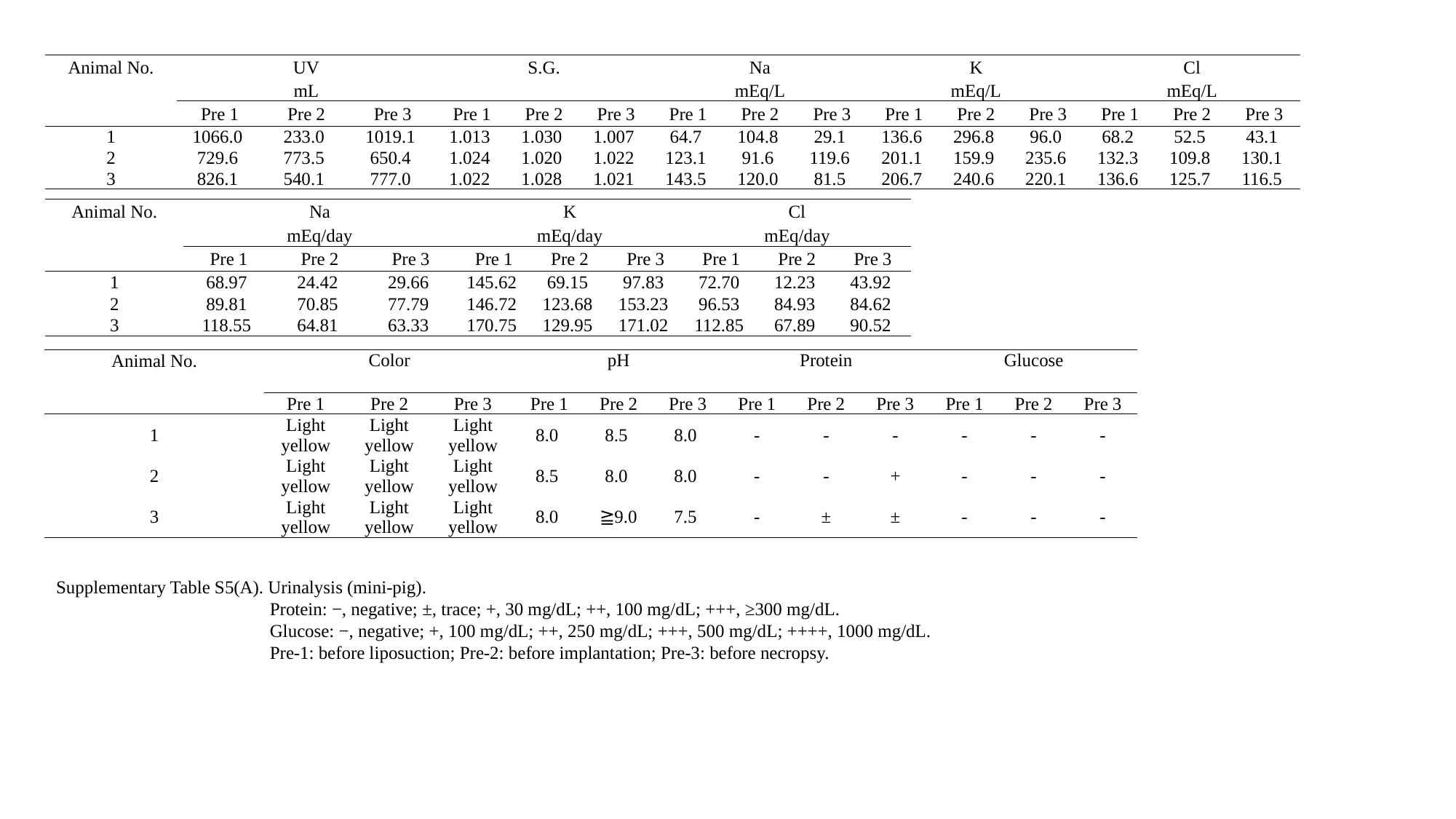

| Animal No. | | UV | | | S.G. | | | Na | | | K | | | Cl | |
| --- | --- | --- | --- | --- | --- | --- | --- | --- | --- | --- | --- | --- | --- | --- | --- |
| | | mL | | | | | | mEq/L | | | mEq/L | | | mEq/L | |
| | Pre 1 | Pre 2 | Pre 3 | Pre 1 | Pre 2 | Pre 3 | Pre 1 | Pre 2 | Pre 3 | Pre 1 | Pre 2 | Pre 3 | Pre 1 | Pre 2 | Pre 3 |
| 1 | 1066.0 | 233.0 | 1019.1 | 1.013 | 1.030 | 1.007 | 64.7 | 104.8 | 29.1 | 136.6 | 296.8 | 96.0 | 68.2 | 52.5 | 43.1 |
| 2 | 729.6 | 773.5 | 650.4 | 1.024 | 1.020 | 1.022 | 123.1 | 91.6 | 119.6 | 201.1 | 159.9 | 235.6 | 132.3 | 109.8 | 130.1 |
| 3 | 826.1 | 540.1 | 777.0 | 1.022 | 1.028 | 1.021 | 143.5 | 120.0 | 81.5 | 206.7 | 240.6 | 220.1 | 136.6 | 125.7 | 116.5 |
| Animal No. | | Na | | | K | | | Cl | |
| --- | --- | --- | --- | --- | --- | --- | --- | --- | --- |
| | | mEq/day | | | mEq/day | | | mEq/day | |
| | Pre 1 | Pre 2 | Pre 3 | Pre 1 | Pre 2 | Pre 3 | Pre 1 | Pre 2 | Pre 3 |
| 1 | 68.97 | 24.42 | 29.66 | 145.62 | 69.15 | 97.83 | 72.70 | 12.23 | 43.92 |
| 2 | 89.81 | 70.85 | 77.79 | 146.72 | 123.68 | 153.23 | 96.53 | 84.93 | 84.62 |
| 3 | 118.55 | 64.81 | 63.33 | 170.75 | 129.95 | 171.02 | 112.85 | 67.89 | 90.52 |
| Animal No. | Color | | | pH | | | Protein | | | Glucose | | |
| --- | --- | --- | --- | --- | --- | --- | --- | --- | --- | --- | --- | --- |
| | | | | | | | | | | | | |
| | Pre 1 | Pre 2 | Pre 3 | Pre 1 | Pre 2 | Pre 3 | Pre 1 | Pre 2 | Pre 3 | Pre 1 | Pre 2 | Pre 3 |
| 1 | Light yellow | Light yellow | Light yellow | 8.0 | 8.5 | 8.0 | - | - | - | - | - | - |
| 2 | Light yellow | Light yellow | Light yellow | 8.5 | 8.0 | 8.0 | - | - | + | - | - | - |
| 3 | Light yellow | Light yellow | Light yellow | 8.0 | ≧9.0 | 7.5 | - | ± | ± | - | - | - |
Supplementary Table S5(A). Urinalysis (mini‑pig).
 Protein: −, negative; ±, trace; +, 30 mg/dL; ++, 100 mg/dL; +++, ≥300 mg/dL.
 Glucose: −, negative; +, 100 mg/dL; ++, 250 mg/dL; +++, 500 mg/dL; ++++, 1000 mg/dL.
 Pre‑1: before liposuction; Pre‑2: before implantation; Pre‑3: before necropsy.

## Slide 6
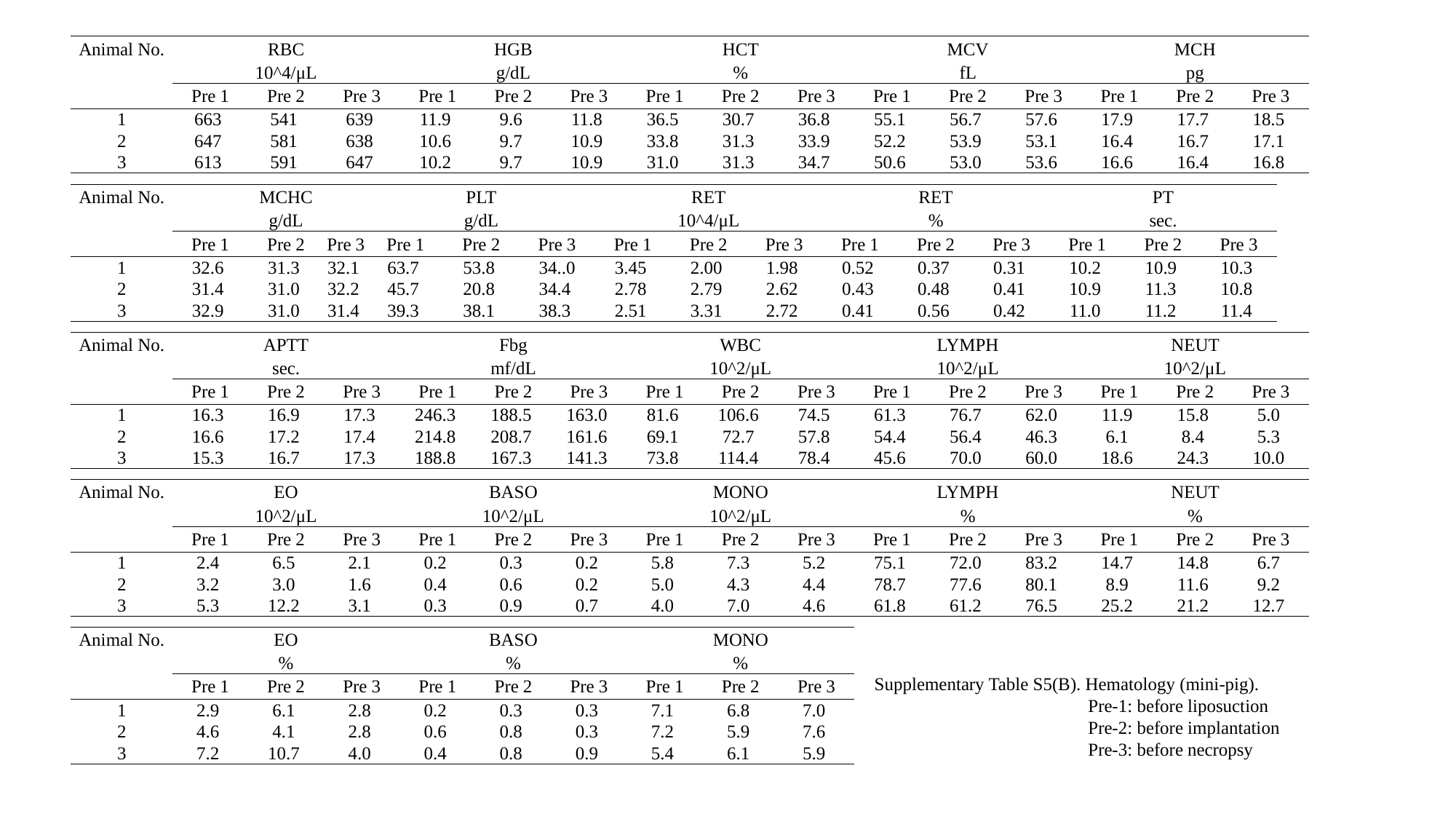

| Animal No. | | RBC | | | HGB | | | HCT | | | MCV | | | MCH | |
| --- | --- | --- | --- | --- | --- | --- | --- | --- | --- | --- | --- | --- | --- | --- | --- |
| | | 10^4/μL | | | g/dL | | | % | | | fL | | | pg | |
| | Pre 1 | Pre 2 | Pre 3 | Pre 1 | Pre 2 | Pre 3 | Pre 1 | Pre 2 | Pre 3 | Pre 1 | Pre 2 | Pre 3 | Pre 1 | Pre 2 | Pre 3 |
| 1 | 663 | 541 | 639 | 11.9 | 9.6 | 11.8 | 36.5 | 30.7 | 36.8 | 55.1 | 56.7 | 57.6 | 17.9 | 17.7 | 18.5 |
| 2 | 647 | 581 | 638 | 10.6 | 9.7 | 10.9 | 33.8 | 31.3 | 33.9 | 52.2 | 53.9 | 53.1 | 16.4 | 16.7 | 17.1 |
| 3 | 613 | 591 | 647 | 10.2 | 9.7 | 10.9 | 31.0 | 31.3 | 34.7 | 50.6 | 53.0 | 53.6 | 16.6 | 16.4 | 16.8 |
| Animal No. | | MCHC | | | PLT | | | RET | | | RET | | | PT | |
| --- | --- | --- | --- | --- | --- | --- | --- | --- | --- | --- | --- | --- | --- | --- | --- |
| | | g/dL | | | g/dL | | | 10^4/μL | | | % | | | sec. | |
| | Pre 1 | Pre 2 | Pre 3 | Pre 1 | Pre 2 | Pre 3 | Pre 1 | Pre 2 | Pre 3 | Pre 1 | Pre 2 | Pre 3 | Pre 1 | Pre 2 | Pre 3 |
| 1 | 32.6 | 31.3 | 32.1 | 63.7 | 53.8 | 34..0 | 3.45 | 2.00 | 1.98 | 0.52 | 0.37 | 0.31 | 10.2 | 10.9 | 10.3 |
| 2 | 31.4 | 31.0 | 32.2 | 45.7 | 20.8 | 34.4 | 2.78 | 2.79 | 2.62 | 0.43 | 0.48 | 0.41 | 10.9 | 11.3 | 10.8 |
| 3 | 32.9 | 31.0 | 31.4 | 39.3 | 38.1 | 38.3 | 2.51 | 3.31 | 2.72 | 0.41 | 0.56 | 0.42 | 11.0 | 11.2 | 11.4 |
| Animal No. | | APTT | | | Fbg | | | WBC | | | LYMPH | | | NEUT | |
| --- | --- | --- | --- | --- | --- | --- | --- | --- | --- | --- | --- | --- | --- | --- | --- |
| | | sec. | | | mf/dL | | | 10^2/μL | | | 10^2/μL | | | 10^2/μL | |
| | Pre 1 | Pre 2 | Pre 3 | Pre 1 | Pre 2 | Pre 3 | Pre 1 | Pre 2 | Pre 3 | Pre 1 | Pre 2 | Pre 3 | Pre 1 | Pre 2 | Pre 3 |
| 1 | 16.3 | 16.9 | 17.3 | 246.3 | 188.5 | 163.0 | 81.6 | 106.6 | 74.5 | 61.3 | 76.7 | 62.0 | 11.9 | 15.8 | 5.0 |
| 2 | 16.6 | 17.2 | 17.4 | 214.8 | 208.7 | 161.6 | 69.1 | 72.7 | 57.8 | 54.4 | 56.4 | 46.3 | 6.1 | 8.4 | 5.3 |
| 3 | 15.3 | 16.7 | 17.3 | 188.8 | 167.3 | 141.3 | 73.8 | 114.4 | 78.4 | 45.6 | 70.0 | 60.0 | 18.6 | 24.3 | 10.0 |
| Animal No. | | EO | | | BASO | | | MONO | | | LYMPH | | | NEUT | |
| --- | --- | --- | --- | --- | --- | --- | --- | --- | --- | --- | --- | --- | --- | --- | --- |
| | | 10^2/μL | | | 10^2/μL | | | 10^2/μL | | | % | | | % | |
| | Pre 1 | Pre 2 | Pre 3 | Pre 1 | Pre 2 | Pre 3 | Pre 1 | Pre 2 | Pre 3 | Pre 1 | Pre 2 | Pre 3 | Pre 1 | Pre 2 | Pre 3 |
| 1 | 2.4 | 6.5 | 2.1 | 0.2 | 0.3 | 0.2 | 5.8 | 7.3 | 5.2 | 75.1 | 72.0 | 83.2 | 14.7 | 14.8 | 6.7 |
| 2 | 3.2 | 3.0 | 1.6 | 0.4 | 0.6 | 0.2 | 5.0 | 4.3 | 4.4 | 78.7 | 77.6 | 80.1 | 8.9 | 11.6 | 9.2 |
| 3 | 5.3 | 12.2 | 3.1 | 0.3 | 0.9 | 0.7 | 4.0 | 7.0 | 4.6 | 61.8 | 61.2 | 76.5 | 25.2 | 21.2 | 12.7 |
| Animal No. | | EO | | | BASO | | | MONO | |
| --- | --- | --- | --- | --- | --- | --- | --- | --- | --- |
| | | % | | | % | | | % | |
| | Pre 1 | Pre 2 | Pre 3 | Pre 1 | Pre 2 | Pre 3 | Pre 1 | Pre 2 | Pre 3 |
| 1 | 2.9 | 6.1 | 2.8 | 0.2 | 0.3 | 0.3 | 7.1 | 6.8 | 7.0 |
| 2 | 4.6 | 4.1 | 2.8 | 0.6 | 0.8 | 0.3 | 7.2 | 5.9 | 7.6 |
| 3 | 7.2 | 10.7 | 4.0 | 0.4 | 0.8 | 0.9 | 5.4 | 6.1 | 5.9 |
Supplementary Table S5(B). Hematology (mini‑pig).
 Pre‑1: before liposuction
 Pre‑2: before implantation
 Pre‑3: before necropsy

## Slide 7
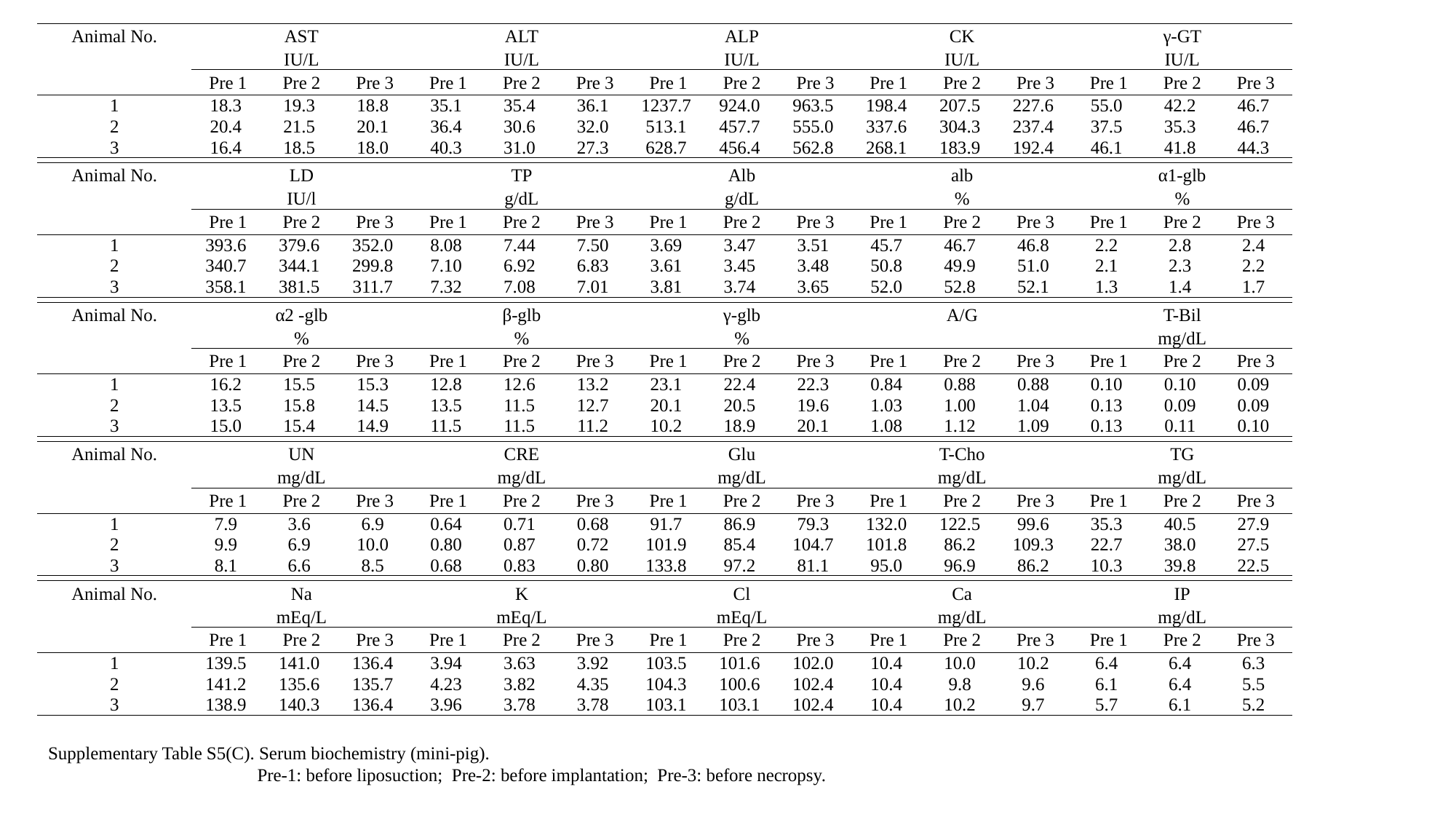

| Animal No. | | AST | | | ALT | | | ALP | | | CK | | | γ-GT | |
| --- | --- | --- | --- | --- | --- | --- | --- | --- | --- | --- | --- | --- | --- | --- | --- |
| | | IU/L | | | IU/L | | | IU/L | | | IU/L | | | IU/L | |
| | Pre 1 | Pre 2 | Pre 3 | Pre 1 | Pre 2 | Pre 3 | Pre 1 | Pre 2 | Pre 3 | Pre 1 | Pre 2 | Pre 3 | Pre 1 | Pre 2 | Pre 3 |
| 1 | 18.3 | 19.3 | 18.8 | 35.1 | 35.4 | 36.1 | 1237.7 | 924.0 | 963.5 | 198.4 | 207.5 | 227.6 | 55.0 | 42.2 | 46.7 |
| 2 | 20.4 | 21.5 | 20.1 | 36.4 | 30.6 | 32.0 | 513.1 | 457.7 | 555.0 | 337.6 | 304.3 | 237.4 | 37.5 | 35.3 | 46.7 |
| 3 | 16.4 | 18.5 | 18.0 | 40.3 | 31.0 | 27.3 | 628.7 | 456.4 | 562.8 | 268.1 | 183.9 | 192.4 | 46.1 | 41.8 | 44.3 |
| Animal No. | | LD | | | TP | | | Alb | | | alb | | | α1-glb | |
| --- | --- | --- | --- | --- | --- | --- | --- | --- | --- | --- | --- | --- | --- | --- | --- |
| | | IU/l | | | g/dL | | | g/dL | | | % | | | % | |
| | Pre 1 | Pre 2 | Pre 3 | Pre 1 | Pre 2 | Pre 3 | Pre 1 | Pre 2 | Pre 3 | Pre 1 | Pre 2 | Pre 3 | Pre 1 | Pre 2 | Pre 3 |
| 1 | 393.6 | 379.6 | 352.0 | 8.08 | 7.44 | 7.50 | 3.69 | 3.47 | 3.51 | 45.7 | 46.7 | 46.8 | 2.2 | 2.8 | 2.4 |
| 2 | 340.7 | 344.1 | 299.8 | 7.10 | 6.92 | 6.83 | 3.61 | 3.45 | 3.48 | 50.8 | 49.9 | 51.0 | 2.1 | 2.3 | 2.2 |
| 3 | 358.1 | 381.5 | 311.7 | 7.32 | 7.08 | 7.01 | 3.81 | 3.74 | 3.65 | 52.0 | 52.8 | 52.1 | 1.3 | 1.4 | 1.7 |
| Animal No. | | α2 -glb | | | β-glb | | | γ-glb | | | A/G | | | T-Bil | |
| --- | --- | --- | --- | --- | --- | --- | --- | --- | --- | --- | --- | --- | --- | --- | --- |
| | | % | | | % | | | % | | | | | | mg/dL | |
| | Pre 1 | Pre 2 | Pre 3 | Pre 1 | Pre 2 | Pre 3 | Pre 1 | Pre 2 | Pre 3 | Pre 1 | Pre 2 | Pre 3 | Pre 1 | Pre 2 | Pre 3 |
| 1 | 16.2 | 15.5 | 15.3 | 12.8 | 12.6 | 13.2 | 23.1 | 22.4 | 22.3 | 0.84 | 0.88 | 0.88 | 0.10 | 0.10 | 0.09 |
| 2 | 13.5 | 15.8 | 14.5 | 13.5 | 11.5 | 12.7 | 20.1 | 20.5 | 19.6 | 1.03 | 1.00 | 1.04 | 0.13 | 0.09 | 0.09 |
| 3 | 15.0 | 15.4 | 14.9 | 11.5 | 11.5 | 11.2 | 10.2 | 18.9 | 20.1 | 1.08 | 1.12 | 1.09 | 0.13 | 0.11 | 0.10 |
| Animal No. | | UN | | | CRE | | | Glu | | | T-Cho | | | TG | |
| --- | --- | --- | --- | --- | --- | --- | --- | --- | --- | --- | --- | --- | --- | --- | --- |
| | | mg/dL | | | mg/dL | | | mg/dL | | | mg/dL | | | mg/dL | |
| | Pre 1 | Pre 2 | Pre 3 | Pre 1 | Pre 2 | Pre 3 | Pre 1 | Pre 2 | Pre 3 | Pre 1 | Pre 2 | Pre 3 | Pre 1 | Pre 2 | Pre 3 |
| 1 | 7.9 | 3.6 | 6.9 | 0.64 | 0.71 | 0.68 | 91.7 | 86.9 | 79.3 | 132.0 | 122.5 | 99.6 | 35.3 | 40.5 | 27.9 |
| 2 | 9.9 | 6.9 | 10.0 | 0.80 | 0.87 | 0.72 | 101.9 | 85.4 | 104.7 | 101.8 | 86.2 | 109.3 | 22.7 | 38.0 | 27.5 |
| 3 | 8.1 | 6.6 | 8.5 | 0.68 | 0.83 | 0.80 | 133.8 | 97.2 | 81.1 | 95.0 | 96.9 | 86.2 | 10.3 | 39.8 | 22.5 |
| Animal No. | | Na | | | K | | | Cl | | | Ca | | | IP | |
| --- | --- | --- | --- | --- | --- | --- | --- | --- | --- | --- | --- | --- | --- | --- | --- |
| | | mEq/L | | | mEq/L | | | mEq/L | | | mg/dL | | | mg/dL | |
| | Pre 1 | Pre 2 | Pre 3 | Pre 1 | Pre 2 | Pre 3 | Pre 1 | Pre 2 | Pre 3 | Pre 1 | Pre 2 | Pre 3 | Pre 1 | Pre 2 | Pre 3 |
| 1 | 139.5 | 141.0 | 136.4 | 3.94 | 3.63 | 3.92 | 103.5 | 101.6 | 102.0 | 10.4 | 10.0 | 10.2 | 6.4 | 6.4 | 6.3 |
| 2 | 141.2 | 135.6 | 135.7 | 4.23 | 3.82 | 4.35 | 104.3 | 100.6 | 102.4 | 10.4 | 9.8 | 9.6 | 6.1 | 6.4 | 5.5 |
| 3 | 138.9 | 140.3 | 136.4 | 3.96 | 3.78 | 3.78 | 103.1 | 103.1 | 102.4 | 10.4 | 10.2 | 9.7 | 5.7 | 6.1 | 5.2 |
Supplementary Table S5(C). Serum biochemistry (mini‑pig).
 Pre‑1: before liposuction; Pre‑2: before implantation; Pre‑3: before necropsy.

## Slide 8
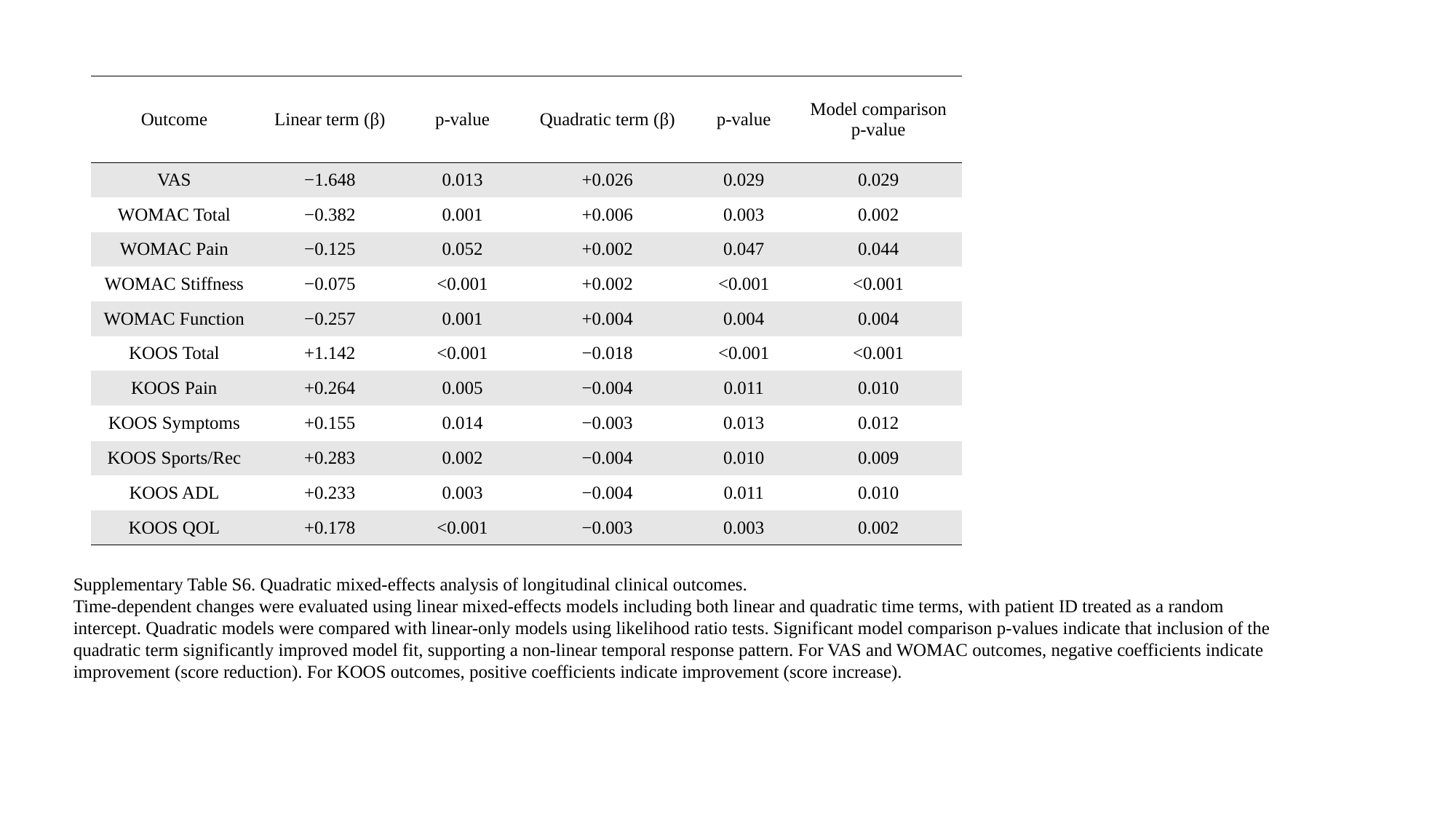

| Outcome | Linear term (β) | p-value | Quadratic term (β) | p-value | Model comparison p-value |
| --- | --- | --- | --- | --- | --- |
| VAS | −1.648 | 0.013 | +0.026 | 0.029 | 0.029 |
| WOMAC Total | −0.382 | 0.001 | +0.006 | 0.003 | 0.002 |
| WOMAC Pain | −0.125 | 0.052 | +0.002 | 0.047 | 0.044 |
| WOMAC Stiffness | −0.075 | <0.001 | +0.002 | <0.001 | <0.001 |
| WOMAC Function | −0.257 | 0.001 | +0.004 | 0.004 | 0.004 |
| KOOS Total | +1.142 | <0.001 | −0.018 | <0.001 | <0.001 |
| KOOS Pain | +0.264 | 0.005 | −0.004 | 0.011 | 0.010 |
| KOOS Symptoms | +0.155 | 0.014 | −0.003 | 0.013 | 0.012 |
| KOOS Sports/Rec | +0.283 | 0.002 | −0.004 | 0.010 | 0.009 |
| KOOS ADL | +0.233 | 0.003 | −0.004 | 0.011 | 0.010 |
| KOOS QOL | +0.178 | <0.001 | −0.003 | 0.003 | 0.002 |
Supplementary Table S6. Quadratic mixed-effects analysis of longitudinal clinical outcomes.
Time-dependent changes were evaluated using linear mixed-effects models including both linear and quadratic time terms, with patient ID treated as a random intercept. Quadratic models were compared with linear-only models using likelihood ratio tests. Significant model comparison p-values indicate that inclusion of the quadratic term significantly improved model fit, supporting a non-linear temporal response pattern. For VAS and WOMAC outcomes, negative coefficients indicate improvement (score reduction). For KOOS outcomes, positive coefficients indicate improvement (score increase).
