# Supplementary material for: Preclinical and early clinical safety of intra-articular spheroid adipose-derived stem cells for knee osteoarthritis: A translational study
Source: Osteoarthr Cartil Open. 2026 Mar 28;8(2):100792. doi: 10.1016/j.ocarto.2026.100792 (PMC13090708; doi:10.1016/j.ocarto.2026.100792)
Supplement: Multimedia component 2 [file mmc2.docx]

Title: Preclinical and Early Clinical Safety of Intra-Articular Spheroid Adipose-Derived Stem Cells for Knee Osteoarthritis: A Translational Study

Authors: Satoshi Sobajima^1,3^*, Yusuke Harada^1^, Tae-sun Kim^1^, Osamu Kisaki^1^, Kaito Otera^1^, Hitoshi Yamauchi^1^, Hiroshi Umemoto^1^, Tomomi Makino^2^, Emiko Nagao^2^, Hideki Iwaguro^1,4^

1 Sobajima Clinic and Medical Institute for Regenerative Medicine, Osaka, Japan
2 NIPPON SHOKUBAI CO., LTD., Osaka, Japan
3 Department of Regenerative Medicine, Kanazawa Medical University, Uchinada, Japan
4 Center for Regenerative Medicine, Kanazawa Medical University, Uchinada, Japan

*Corresponding author: Satoshi Sobajima, MD, PhD
Sobajima Clinic and Medical Institute for Regenerative Medicine, 2-2-6 Aramotokita, Higashiosaka, Osaka 577-0011, Japan
E-mail: [orthohealing@soba-cli.com](mailto:orthohealing@soba-cli.com)

Abstract

Objective: To evaluate preclinical and early clinical safety of intra-articular spheroid adipose-derived stem cells (S-ADSCs) for knee osteoarthritis and to summarize exploratory outcomes.

Methods: This program included a minipig study (n=3; Day 31) and a clinical cohort (n=5; Week 52). Each knee received 42,000 spheroids (500 cells/spheroid; 2.1×10⁷ cells) in 5 mL. Preclinical assessments included clinical monitoring, laboratory tests, necropsy, and H&E histology of distal femur, proximal tibia, and medial/lateral menisci. In patients, adverse events and serious adverse events were captured through Week 52; exploratory outcomes included pain (visual analog scale [VAS]) and function (KOOS and WOMAC). Exploratory quadratic mixed-effects models assessed non-linear time trends.

Results: Minipigs showed no abnormal clinical signs, laboratory changes, or treatment-related findings at necropsy or on knee histology through Day 31. All five patients completed 52-week follow-up; no treatment-related adverse events or serious adverse events, infections, hemarthroses, or acute post-injection flares requiring medical treatment occurred. Exploratory outcomes were heterogeneous: two participants showed sustained improvement through Week 52, whereas the remaining three showed non-sustained patterns; two improved early with partial return toward baseline, and one experienced transient pain worsening at Week 4 without objective inflammation or infection followed by later recovery. Quadratic models suggested early improvement with later attenuation.

Conclusion: Intra-articular S-ADSC spheroids at this dose demonstrated acceptable safety in a large-animal model and a first-in-human cohort, supporting further controlled studies.

Trial registration: Japan Registry of Clinical Trials (jRCTb050200097; first public release December 17, 2020).

Keywords: knee osteoarthritis; adipose-derived stem cells; spheroid; intra-articular injection; safety; first‑in‑human

Introduction

Osteoarthritis (OA) is a chronic, degenerative joint disease characterized by progressive cartilage breakdown, pain, and functional impairment and represents a growing global burden in aging societies [1,2]. Clinical management is typically stepwise and includes education and exercise-based therapy, pharmacological treatment, and intra-articular injections, with total knee arthroplasty as the definitive option for end-stage disease; however, available options mainly provide symptomatic relief and do not reliably modify disease progression [1,3]. Therefore, there remains an unmet need for less invasive therapies with the potential to improve symptoms while maintaining a favorable safety profile.

Adipose-derived stem cells (ADSCs) are an attractive cell source for regenerative medicine because adipose tissue can be harvested with relative ease and yields a higher number of progenitor cells than bone marrow [4–6]. ADSCs are also reported to exert anti-inflammatory and immunomodulatory effects and to secrete trophic factors that may support tissue repair [4–7]. Clinical experience with adipose-based approaches for knee OA includes stromal vascular fraction (SVF), micro-fragmented adipose tissue (MFAT), and culture-expanded ADSC preparations. Several early studies have reported feasibility and symptom changes after intra-articular delivery of mesenchymal stromal/stem cell products in knee OA, but product composition, handling, and manufacturing processes vary widely across studies [8–14]. This heterogeneity makes cross-study comparison difficult and highlights the importance of standardized product formats with careful safety evaluation.

Spheroid ADSCs (S‑ADSCs) have emerged as a rational delivery format. Compared with conventional two‑dimensional cultures, three‑dimensional aggregation can enhance cell–cell interactions, stress resistance, and paracrine activity, with anti‑inflammatory effects reported in vitro and in vivo [15–17]. At the same time, spheroid biology can be influenced by oxygen gradients; hypoxia‑related signaling may support trophic factor secretion but requires attention to spheroid size and culture conditions [19,20]. In this program, S‑ADSCs were assembled using a mildly cell‑adhesive multicavity platform to promote uniform aggregation and gentle, enzyme‑free harvesting, aiming to support consistent processing for intra‑articular administration [21,22].

Against this backdrop, we designed a translational, safety‑focused program comprising a preclinical large‑animal step and an early clinical step for symptomatic knee OA. We hypothesized that intra‑articular administration of standardized S‑ADSCs would be locally and systemically well tolerated. Here, we report safety outcomes in both settings and summarize exploratory patient‑reported outcomes. The clinical component was conducted under Japan’s Act on the Safety of Regenerative Medicine (ASRM) [23] and registered with the Japan Registry of Clinical Trials (jRCTb050200097) [24].

Materials and Methods

Study overview

This translational program comprised (i) a preclinical large-animal safety study and (ii) an early clinical study focused on safety after intra-articular administration of spheroid adipose-derived stem cells (S-ADSCs) in symptomatic knee osteoarthritis.

Preclinical study

Animals and ethical approval

Eight-month-old female Nippon Institute for Biological Science (NIBS) miniature pigs were housed under controlled conditions (temperature 20-28°C; humidity 30-80%; 12-hour light-dark cycle) and fed a commercial diet with water ad libitum. The protocol was approved by the Animal Experiment Committee of Japan Bio Research Center Co., Ltd. (approval date: November 12, 2018; approval number: 380293).

Fat harvesting and cell isolation

Under general anesthesia and povidone-iodine skin preparation, subcutaneous adipose tissue was harvested bilaterally using tumescent infiltration (500 mL normal saline + 20 mL 2% lidocaine) and liposuction cannula aspiration to obtain 80-100 g of adipose tissue. The tissue was washed and enzymatically digested on the Celution 800/CRS system with Celase GMP, and cells were recovered by serial centrifugation and washing for later expansion.

Cell expansion and spheroid fabrication

Cell suspensions were centrifuged (220 x g, 5 min) and washed twice with PBS. Pellets were resuspended in ADSC-2-based medium (5.0% FBS) and seeded into T175 flasks (37°C, 5% CO2). For passaging, cells were washed with PBS, detached with TrypLE, and replated into T525 flasks at 5,000 cells/cm² with medium changes every 2-3 days until 80-90% confluence. Cells were cryopreserved in 10% DMSO and stored in liquid nitrogen until use; number and viability were measured by an automated cell counter.

ADSC spheroids were assembled on mildly cell-adhesive multicavity plates (MicoCell). Cavities were prefilled with PBS and gently pipetted to remove bubbles, then filled with serum-free ADSC-2 and incubated overnight (Day 1; 37°C, 5% CO2). On Day 2, ADSCs were seeded at 500 cells per cavity (21 mL of 1.0 x 10⁶ cells/mL) and incubated for 72 h (Days 2-5). Spheroids were harvested by removing the medium, resuspending in PBS, rinsing twice with Ringer's lactate, and suspending in 5 mL Ringer's lactate for administration.

All preclinical cell processing was performed in a clinic-affiliated research laboratory at Sobajima Clinic and the Medical Institute for Regenerative Medicine (Osaka, Japan) operated at Biosafety Level 2. All open manipulations were conducted inside certified Class II biological safety cabinets. This laboratory is physically separated from the clinical cell processing center (CPC), with dedicated equipment/consumables and routine chemical/UV decontamination between campaigns.

Timeline of preparation

The MicoCell plates were preconditioned and de-bubbled and then left overnight (Day 1). On the next day (Day 2), ADSCs were seeded at 500 cells per cavity and incubated for 72 h (Days 2–5) to assemble spheroids. On Day 5 (72 h after seeding), spheroids were harvested and administered intra-articularly on the same day. The final product was transported in sterile, sealed containers to the animal housing facility (Japan Bio Research Center Co., Ltd.) and injected on the day of harvest.

Release testing and dose rationale

Before administration, each lot underwent release testing according to predefined standard operating procedure (SOP) acceptance criteria, including sterility testing (aerobic and anaerobic cultures), bacterial endotoxin testing by limulus amebocyte lysate (LAL) assay, mycoplasma testing (culture/PCR per SOP), and before-release viability assessment using an automated cell counter (Supplementary Table S1). Only lots meeting all acceptance criteria were released for use.

The intra-articular dose per knee consisted of 42,000 spheroids (500 cells per spheroid; total cell content 2.1 x 10⁷ cells) suspended in 5 mL Ringer's lactate. This dose was selected based on (i) the manufacturing capacity of a single 42,000-cavity plate enabling a standardized, reproducible dose per knee, (ii) a clinically practical injection volume for intra-articular delivery, and (iii) consistency with cell doses previously used for intra-articular administration of culture-expanded ADSCs in early-phase studies. The same dose and volume were used for the preclinical and clinical steps to support translational comparability.

S-ADSC administration and peri-procedural care

Under fluoroscopic guidance (C-arm), needle position was confirmed within the knee joint cavity and S-ADSCs were injected intra-articularly. To reduce the risk of procedure-related infection after joint puncture and cell administration, ampicillin sodium (1 g/animal/day) was administered intramuscularly for three days according to the contracted animal facility's standard prophylactic regimen. Buprenorphine hydrochloride (0.01 mg/kg) was administered intramuscularly once daily for seven days for analgesia.

Safety assessments and histopathology (including injected knee)

Safety was evaluated by urinalysis, hematology, and serum biochemistry at three time points: baseline at fat harvesting, at transplantation, and pre-necropsy. On Day 31, necropsy was performed to measure organ weights (relative to body weight) and to conduct histopathology. Organs/tissues were fixed (lungs infused before fixation; eyeballs fixed in glutaraldehyde-formalin and then in neutral-buffered formalin); femur and sternum samples were decalcified and paraffin-embedded. All sections were H&E-stained.

To assess local joint tolerability at the experimental endpoint, representative tissues from the injected knee joint were also processed for H&E staining, including distal femur (articular cartilage), proximal tibia (articular cartilage), and medial and lateral menisci.

Early clinical study

Trial registration and ethics

The single-arm clinical study was conducted under Japan's ASRM with prior review by the Certified Committee for Regenerative Medicine (CCRM) at the Japanese Association for the Promotion of State-of-the-Art in Medicine (JAPSAM) (approval No. 344; June 1, 2020). The study was registered in the Japan Registry of Clinical Trials (jRCTb050200097; first public release December 17, 2020). All participants provided written informed consent.

Patients

Five patients with knee osteoarthritis were enrolled. Participants attended scheduled hospital visits for supervised rehabilitation and were instructed in a standardized home exercise program. Full inclusion and exclusion criteria are provided in Supplementary Table S2.

Liposuction procedure

Approximately 10 mL of subcutaneous adipose tissue was obtained by mini-liposuction under sterile conditions using local tumescent anesthesia. Adipose tissue was harvested from the lower back and buttock (lumbar–gluteal) region using a small multiport cannula connected to a sterile syringe. Harvested tissue was rinsed with Ringer's lactate immediately after collection.

Isolation of ADSCs and spheroid culture

Tissue was enzymatically digested using GMP-grade collagenase/thermolysin (37°C, 30 min with gentle agitation), centrifuged (220 x g, 5 min), and washed twice with PBS. Cells were resuspended in ADSC-2 medium supplemented with 2.5% autologous serum and seeded into T175 flasks (37°C, 5% CO2). For passaging, cells were washed with PBS, detached with TrypLE, and plated into T525 flasks at 5,000 cells/cm² with medium changes every 2-3 days until 80-90% confluence. Cells were cryopreserved in 10% DMSO and stored in liquid nitrogen until use; counts and viability were measured by an automated cell counter.

Spheroid fabrication followed the MicoCell workflow described above: plates with 42,000 cavities were pretreated to remove bubbles, filled with serum-free medium, and ADSCs were seeded at 500 cells/cavity (21 mL of 1.0 x 10⁶ cells/mL) and cultured for 3 days. The spheroids were harvested into PBS, rinsed twice with Ringer's lactate, and suspended in 5 mL Ringer's lactate for injection. Clinical-grade ADSC expansion and spheroid assembly were performed in the on-site CPC at Sobajima Clinic and the Medical Institute for Regenerative Medicine (Osaka, Japan) under an ASRM-compliant quality system aligned with GCTP principles.

Timeline of Preparation

Clinical processing matched the preclinical workflow: Day 1 for plate preconditioning/de‑bubbling, Days 2–5 for the 72 h spheroid assembly, and intra‑articular administration on Day 5 (same day as assembly completion). Adipose tissue was harvested at the clinic and processed and spheroidized in the on‑site CPC following the 5‑day workflow, followed by Day 5 intra‑articular administration.

Quality control and dose

Clinical lots underwent release testing according to SOP acceptance criteria (Supplementary Table S1). The intra-articular dose per knee consisted of 42,000 spheroids (500 cells per spheroid; total content 2.1 x 10⁷ cells) suspended in 5 mL Ringer's lactate.

Intra-articular administration

Intra-articular injection into the target knee was performed under ultrasound guidance using a standard superolateral approach (suprapatellar recess) with a 21G needle. When joint effusion was present, aspiration was performed before administration. No systemic anesthesia was used.

Safety and exploratory endpoints

The primary endpoint was safety, defined as the incidence, severity, and course of adverse events and serious adverse events from fat harvesting through Week 52. Adverse events were graded according to CTCAE v5.0 [25], and seriousness followed ICH definitions [26]. Relatedness was assessed by the investigator.

Exploratory assessments included pain measured by the visual analog scale (VAS; 0–100; higher scores indicate worse pain), function measured by the Knee injury and Osteoarthritis Outcome Score (KOOS; normalized to 0–100; higher scores indicate better status), and the Western Ontario and McMaster Universities Osteoarthritis Index (WOMAC; raw scores in the original direction; higher scores indicate worse status). Clinical evaluations were scheduled at baseline and Weeks 4, 12, 26, and 52 after treatment.

MRI-based structural evaluations

As a prespecified exploratory endpoint, knee MRI was acquired at baseline and Week 52 and archived for analysis outside the scope of this safety-focused manuscript.

Statistical analysis

Safety outcomes were summarized descriptively, and exploratory outcomes were displayed as individual trajectories. To quantitatively describe non-linear time trends in patient-reported outcomes, we performed exploratory longitudinal analyses using linear mixed-effects models with a random intercept for participant and fixed effects for week and week². Quadratic models were compared with linear models using likelihood ratio tests (df=1). Coefficient p values and 95% confidence intervals were computed using a t distribution with df=20, and results are summarized in Supplementary Table S6. No imputation was performed. As an illustrative planning exercise, an example sample-size calculation for a future two-arm randomized trial was performed (see Discussion).

Results

Spheroid formation and product release

ADSC spheroids formed uniformly on the MicoCell plate (42,000 cavities) within 72 hours at 500 cells/cavity (Fig. 1A, B). Spheroids exhibited compact morphology without satellite aggregates; the size distribution centered around 150 ± 20 µm. Pre‑release viability exceeded 90% for all lots. Release testing (sterility by aerobic/anaerobic culture, endotoxin by LAL assay within acceptance limits, mycoplasma by culture/PCR) met SOP criteria for all lots (Supplementary Table S1).

Preclinical safety (minipig)

All minipigs survived to Day 31 without abnormal clinical signs, and body weight remained stable. Urinalysis, hematology, and serum biochemistry did not show clinically meaningful abnormalities across baseline, transplantation, and pre-necropsy time points. At necropsy, organ weights were within expected ranges, and histopathology of major organs/tissues showed no treatment-related findings (Table 1). Detailed body weight trajectories, relative organ weights, and laboratory results are provided in Supplementary Tables S3–S5.

At the injected knee joints, representative H&E-stained sections of distal femur articular cartilage, proximal tibia articular cartilage, and medial and lateral menisci showed preserved architecture without apparent inflammatory infiltrates or ectopic tissue reactions (Fig. 2).

Clinical safety (n=5)

Baseline characteristics are summarized in Table 2. All five patients completed 52-week follow-up. No treatment-related adverse events or serious adverse events were observed through Week 52 (Table 3), and no infections or hemarthroses occurred. No acute post-injection inflammatory flares requiring medical treatment were reported. One participant experienced transient worsening of pain at Week 4 without objective signs of inflammatory flare or infection and improved at subsequent visits.

Visual appearance and handling of the syringe suspension

Immediately before IA administration, the final product comprised 42,000 spheroids freely suspended in 5 mL of Ringer’s lactate (Fig. 1C). After gentle inversion (2–3 times), the suspension mixed evenly without foam generation; no macroscopic aggregates (greater than 1 mm), precipitation, or stringing were seen. The suspension passed smoothly through the bore of a standard sterile syringe without plunger resistance, leakage, or needle blockage. The photograph was captured in the cell‑processing facility with no patient or site identifiers, and no selective photo enhancement or alteration was performed.

Exploratory patient-reported outcomes

Individual trajectories for pain and function are shown in Fig. 3 (VAS) and Fig. 4A–B (KOOS and WOMAC). Exploratory outcomes were heterogeneous. For pain (VAS), two participants showed sustained reductions through Week 52, whereas the remaining three demonstrated non-sustained patterns (two with early reductions and partial return toward baseline; one with transient worsening at Week 4 without objective signs of inflammatory flare or infection and subsequent recovery) (Fig. 3). KOOS (Fig. 4A) and WOMAC (Fig. 4B) scores showed similar inter-individual heterogeneity across domains.

Exploratory quadratic mixed-effects modeling (random intercept for participant) suggested non-linear time trends in patient-reported outcomes; model comparison by likelihood ratio testing generally favored inclusion of a quadratic term for VAS and selected KOOS/WOMAC scales (Supplementary Table S6). Given the small, uncontrolled cohort (n=5), these analyses are hypothesis-generating.

Discussion

The most important finding of this study was that intra-articular administration of spheroid adipose-derived stem cells (S‑ADSCs) was feasible and was not associated with treatment-related safety signals in a large-animal model through Day 31 or in an initial human cohort through Week 52. Within a translational, safety-first framework, these data support that a standardized spheroid format can be manufactured to predefined release criteria and delivered intra‑articularly at the tested dose and volume without clinically meaningful local or systemic toxicity.

In the preclinical study, no abnormal clinical signs, clinically meaningful laboratory changes, or treatment-related pathological findings were observed at the experimental endpoint. Importantly, local joint tolerability was assessed directly at necropsy: representative H&E-stained sections of distal femur articular cartilage, proximal tibia articular cartilage, and medial and lateral menisci showed preserved tissue architecture without apparent inflammatory infiltrates or ectopic tissue reactions. Although the minipig knees were not an OA disease model, inclusion of endpoint knee histology strengthens interpretation of local safety for intra-articular delivery of this spheroid preparation.

In the clinical cohort (n=5), no treatment-related adverse events or serious adverse events occurred through Week 52, and no infections or hemarthroses were reported. No acute post-injection inflammatory flares requiring medical treatment were observed. One participant experienced transient worsening of pain at Week 4 without objective signs of inflammatory flare or infection and improved thereafter. Such short-term symptom fluctuation can occur in symptomatic knee OA and highlights the importance of controlled designs and adequate follow-up when interpreting patient-reported outcomes in early-phase studies.

Spheroids were chosen because three-dimensional aggregation can strengthen paracrine and anti-inflammatory programs and may help cells withstand mechanical stress and hypoxia in the intra-articular environment. Bartosh and colleagues reported that mesenchymal stromal cell spheroids up-regulate anti-inflammatory mediators and show stronger anti-inflammatory effects than two-dimensional monolayers [15]. A review by Cesarz and Tamama also summarized improved survival and secretome profiles in spheroids. We consider these features relevant for the intra-articular niche [18].

Operationally, our process used a mildly cell-adhesive multicavity plate (MicoCell) to generate uniform spheroids within 72 hours and to enable gentle, enzyme-free harvesting. Release viability exceeded 90%. We seeded 500 cells per cavity across 42,000 cavities and produced 42,000 spheroids per knee in 5 mL of Ringer’s lactate, supporting reproducibility and providing clear lot-release criteria for the spheroid format [22]. This per-knee dose was selected primarily to be conservative for first-in-human use and to keep manufacturing simple and stable while maintaining a clinically practical injection volume.

Although the clinical component was not designed to evaluate efficacy, exploratory patient-reported outcomes showed heterogeneous trajectories across individuals. Two participants exhibited sustained improvement through Week 52, whereas three showed non-sustained patterns, including one with transient worsening at Week 4 followed by recovery. Consistent with these patterns, exploratory quadratic mixed-effects modeling suggested non-linear time trends for VAS and selected KOOS/WOMAC scales, compatible with early improvement and partial attenuation at later time points (Supplementary Table S6). Given the small, uncontrolled cohort and multiple exploratory endpoints, these analyses should be interpreted as hypothesis-generating rather than confirmatory.

Our study was not designed for head-to-head comparisons, but the absence of immediate flares and the practical injectability are consistent with good local tolerability. It is possible that spheroids reduce early reactogenicity compared with single-cell suspensions; however, this requires direct testing in randomized studies. To the best of our knowledge, the clinical component represents an early first-in-human report of intra-articular administration of standardized ADSC spheroids for knee osteoarthritis. Efficacy should be tested and confirmed in subsequent controlled trials. As a next step, a planned expansion study will evaluate a higher total cell content (approximately 84,000 spheroids; 4.2 × 10⁷ cells) while keeping the per-spheroid cell number and injection volume constant, to explore potential dose dependence and to confirm injectability and safety margins. Doses in this range have been administered in prior intra‑articular studies of monolayer‑expanded ADSCs without unexpected short‑term safety concerns [8,11]. In addition, meta‑analytic evidence from randomized trials and dose‑comparison studies in adipose‑based products suggests that clinical and/or imaging responses may vary by dose, although cross‑study heterogeneity is substantial [12,13]. Therefore, we intend to conduct a prospective dose‑ranging study with CTCAE v5.0 safety surveillance and predefined stopping rules, using common endpoints (VAS, KOOS, WOMAC, and MRI) [25,26].

To inform future trial design, we performed an illustrative power and sample-size exploration based on the observed VAS change and variability in this cohort (mean Δ=20.2, SD=27.6). With n=5, the estimated achieved power is approximately 20%, reinforcing that clinical outcome signals are exploratory. For planning purposes, a two-arm randomized controlled trial (two-sided α=0.05; 80% power; equal allocation) targeting a between-group difference of 20.2 points on a 0–100 VAS would require approximately 30 participants per group, whereas assuming a more conservative 10-point difference would increase the requirement to approximately 120 participants per group. These calculations are intended for planning and should be refined using clinically meaningful effect sizes and anticipated attrition.

We have several limitations in this study. First, the clinical study was a small, single-arm cohort (n=5), which limits generalizability and precludes definitive conclusions regarding efficacy. Second, the preclinical step had a limited cohort size and follow-up duration (Day 31) and was conducted in non-diseased joints; longer-term evaluation and OA disease models would further inform safety and local tissue responses. Third, while endpoint knee histology was performed, assessment was limited to representative tissues and routine H&E staining; more comprehensive joint evaluation (e.g., synovium, standardized histologic scoring, and additional stains) would strengthen structural interpretation. Fourth, MRI was acquired as a prespecified exploratory endpoint but was not analyzed within this safety-focused report to avoid selective outcome reporting; it will be evaluated in a larger efficacy-oriented study with adequate power. Finally, mechanistic interpretation relies on prior literature for spheroid biology, and in-house functional assays were intentionally limited to product characterization, which is appropriate for a safety-first program but restricts mechanistic conclusions.

Conclusions

Intra-articular administration of S-ADSCs showed acceptable safety in both preclinical and early clinical settings. These findings support further controlled, adequately powered studies to establish efficacy and to optimize dosing, patient selection, and imaging/biomarker endpoints in knee OA.

The Translational Potential of This Article

This study suggests that intra‑articular administration of S‑ADSCs, prepared by a standardized 5‑day workflow and delivered at a fixed spheroid dose, is locally and systemically tolerable in a large‑animal model and an early human cohort. These findings support the design of future controlled, dose‑ranging trials to test clinical efficacy and to refine indications for knee OA.

Acknowledgments

We thank the staff of Japan Bio Research Center Co., Ltd. for animal care and necropsy support and the clinical rehabilitation team for patient follow‑up. We also acknowledge general technical discussions regarding MicoCell with employees of NIPPON SHOKUBAI CO., LTD. Final study design, data collection, data analysis, interpretation, and the decision to submit were the responsibility of the authors. We also thank Brian C. Nolan for his professional medical English editing.

Funding

Preclinical (animal) work was funded by NIPPON SHOKUBAI CO., LTD. The early clinical study was jointly funded by Sobajima Clinic and NIPPON SHOKUBAI CO., LTD.; participants were not charged for the investigational treatment. No external grants were received. MicoCell plates were kindly supplied by NIPPON SHOKUBAI CO., LTD.; the company as an institution had no role in study design, conduct, data analysis, interpretation, or manuscript preparation.

Author Contributions (CRediT taxonomy)

Conceptualization: Satoshi Sobajima; Methodology: Yusuke Harada; Investigation: Tae-sun Kim; Formal analysis: Hitoshi Yamauchi, Hiroshi Umemoto; Resources: Kaito Otera; Data curation: Osamu Kisaki; Writing – original draft: Satoshi Sobajima; Writing – review & editing: all authors; Visualization: Yusuke Harada; Supervision: Hideki Iwaguro; Project administration: Hideki Iwaguro; Funding acquisition: Satoshi Sobajima, Tomomi Makino.

Ethics Approval and Trial Registration

The clinical study was conducted under Japan’s Act on the Safety of Regenerative Medicine with prior review by the Certified Committee for Regenerative Medicine (CCRM) at the Japanese Association for the Promotion of State‑of‑the‑Art in Medicine (JAPSAM) (approval No. 344; 1 June 2020). The study was registered on the Japan Registry of Clinical Trials (jRCTb050200097; first public release December 17, 2020). All participants provided written informed consent.

Data Availability

De‑identified individual participant data and the statistical analysis plan will be available from the corresponding author upon reasonable request and subject to institutional policies and participant consent. The protocol synopsis is available on the Japan Registry of Clinical Trials (jRCTb050200097).

Competing Interests

Tomomi Makino and Emiko Nagao are employees of NIPPON SHOKUBAI CO., LTD., which funded the preclinical work, jointly funded the early clinical study, and supplied MicoCell plates. The other authors declare no competing interests.

Declaration of generative AI in scientific writing

During the preparation of this work, the authors used ChatGPT (OpenAI) to assist with language editing and improving readability. After using this tool, the authors reviewed and edited the content as needed and take full responsibility for the content of this publication.

Figure legend

Figure 1. Spheroid formation using the MicoCell plate and the final syringe product.

(A) Photograph of the 42,000‑cavity MicoCell plate; approximately 2.1×10⁷ ADSCs are seeded to form 42,000 spheroids (500 cells/spheroid).

(B) Microscopic images illustrating spheroid formation within 72 h (top: NIBS minipig; bottom: patient).

(C) Representative syringe containing the final S‑ADSC suspension (42,000 spheroids in 5 mL Ringer’s lactate) immediately before intra‑articular injection; spheroids are visible as fine floating particulates.

Figure 2. Representative H&E-stained sections of injected minipig knee joints at Day 31. (A) Distal femoral condyle (articular cartilage). (B) Proximal tibial plateau (articular cartilage). (C) Medial meniscus. (D) Lateral meniscus. Scale bars: 1 mm.

Figure 3. Individual pain trajectories on the Visual Analog Scale (VAS). Each line represents one patient (n=5). Visits: baseline and Weeks 4, 12, 26, and 52. Higher scores indicate worse pain.

Figure 4. Individual trajectories of function outcomes (KOOS and WOMAC).

(A) KOOS total and subscales (Pain, Symptoms, ADL, Sports/Rec, QOL) normalized to a 0–100 scale at baseline and Weeks 4, 12, 26, and 52 (higher scores indicate better status).

(B) WOMAC total and subscales (Pain, Stiffness, Function) shown as raw scores at the same visits (original direction: higher scores indicate worse status).

Tables

Table 1. Preclinical safety in NIBS minipigs: (A) daily clinical observations through Day 31 and (B) necropsy summary at Day 31.

(A) Animal‑level observation grid across study days (0–31); ‘N’ denotes normal.

(B) Macroscopic and histopathology of major organs/tissues and target sites; no treatment‑related lesions were identified.

Table 2. Baseline characteristics of the clinical cohort (n = 5): sex, age, affected side, Kellgren–Lawrence (K–L) grade, height, weight, and body mass index (BMI). Data are presented as mean ± SD or n (%) unless otherwise indicated.

Table 3. Adverse events through Week 52 (n=5).

Events are coded and graded according to CTCAE v5.0; seriousness is defined per ICH E2A. Relationship to study treatment was assessed by the investigator. No treatment‑related AEs or SAEs were observed, and no immediate intra‑articular flares (swelling/pain), infections, hemarthrosis, or hospitalizations occurred. Counts reflect observed cases only (no imputation). AE, adverse event; SAE, serious adverse event; CTCAE, Common Terminology Criteria for Adverse Events; ICH, International Council for Harmonisation.

Supplementary Tables

Table S1. Lot‑specific clinical product release testing for S‑ADSC spheroids. Each lot was tested for sterility, endotoxin (LAL assay), mycoplasma, and pre‑injection viability (%), and all lots met SOP acceptance criteria.

Table S2. Full inclusion and exclusion criteria for the clinical safety study (detailed listing).

Table S3. Minipig body weight trajectories until Day 31.

Table S4. Minipig relative organ weights at necropsy (Day 31).

Table S5. Minipig summary at Day 31 following intra‑articular S‑ADSC administration. Findings include urinalysis.

Table S6. Quadratic mixed-effects analysis of longitudinal clinical outcomes.

Time-dependent changes were evaluated using linear mixed-effects models including both linear and quadratic time terms, with patient ID treated as a random intercept. Quadratic models were compared with linear-only models using likelihood ratio tests. Significant model comparison p-values indicate that inclusion of the quadratic term significantly improved model fit, supporting a non-linear temporal response pattern. For VAS and WOMAC outcomes, negative coefficients indicate improvement (score reduction). For KOOS outcomes, positive coefficients indicate improvement (score increase).

References

1. Felson DT, Lawrence RC, Dieppe PA, Hirsch R, Helmick CG, Jordan JM, et al. Osteoarthritis: new insights. Ann Intern Med. 2000;133(8):635–646.

2. Cross M, Smith E, Hoy D, Nolte S, Ackerman I, Fransen M, et al. The global burden of hip and knee osteoarthritis: estimates from the Global Burden of Disease 2010 study. Ann Rheum Dis. 2014;73(7):1323–1330.

3. Diekman BO, Guilak F. Stem cell‑based therapies for osteoarthritis: challenges and opportunities. Curr Opin Rheumatol. 2013;25(1):119–126.

4. Gimble JM, Katz AJ, Bunnell BA. Adipose‑derived stem cells for regenerative medicine. Circ Res. 2007;100(9):1249–1260.

5. Mizuno H, Tobita M, Uysal AC. Concise review: adipose‑derived stem cells as a novel tool for future regenerative medicine. Stem Cells. 2012;30(5):804–810.

6. Zuk PA, Zhu M, Ashjian P, De Ugarte DA, Huang JI, Mizuno H, et al. Human adipose tissue is a source of multipotent stem cells. Mol Biol Cell. 2002;13(12):4279–4295.

7. Pittenger MF, Discher DE, Péault BM, Phinney DG, Hare JM, Caplan AI. Mesenchymal stem cell perspective: cell biology to clinical progress. NPJ Regen Med. 2019;4:22.

8. Jo CH, Lee YG, Shin WH, Kim H, Chai JW, Jeong EC, et al. Intra‑articular injection of mesenchymal stem cells for the treatment of osteoarthritis of the knee: a proof‑of‑concept clinical trial. Stem Cells. 2014;32(5):1254–1266.

9. Kim YS, Choi YJ, Koh YG. Mesenchymal stem cell implantation in knee osteoarthritis: evidence from randomized controlled trials. Am J Sports Med. 2015;43(9):2293–2301.

10. Orozco L, Munar A, Soler R, Alberca M, Soler F, Huguet M, et al. Treatment of knee osteoarthritis with autologous mesenchymal stem cells: a pilot study. Transplantation. 2013;95(12):1535–1541.

11. Lee WS, Kim HJ, Kim KI, Kim GB, Jin W. Intra‑articular injection of autologous adipose tissue‑derived mesenchymal stem cells for the treatment of knee osteoarthritis: a phase IIb, randomized, placebo‑controlled clinical trial. Stem Cells Transl Med. 2019;8(6):504–511.

12. Kim KI, Kim MS, Kim JH. Intra‑articular injection of autologous adipose‑derived stem cells or stromal vascular fraction for knee osteoarthritis: a meta‑analysis of randomized trials. Am J Sports Med. 2023;51(10):2601–2614.

13. Tsubosaka M, Matsui M, Ueda S, Yabuuchi K, Sakamoto A, Sekiya I. Comparison of clinical and imaging outcomes of different doses of adipose‑derived stromal vascular fraction cell treatment for knee osteoarthritis. Cell Transplant. 2021;30:09636897211067454.

14. Chen CF, Hu CC, Wu CT, Hong CJ, Lai PL, Chen MH, et al. Treatment of knee osteoarthritis with intra‑articular injection of allogeneic adipose‑derived stem cells (ELIXCYTE®): a phase I/II randomized, active‑control clinical trial. Stem Cell Res Ther. 2021;12(1):262.

15. Bartosh TJ, Ylöstalo JH, Mohammadipoor A, Bazhanov N, Coble K, Claypool K, et al. Aggregation of human mesenchymal stromal cells (MSCs) into 3D spheroids enhances their anti‑inflammatory properties. Proc Natl Acad Sci U S A. 2010;107(31):13724–13729.

16. Baraniak PR, McDevitt TC. Scaffold‑free culture of mesenchymal stem cell spheroids in suspension preserves multilineage potential. Cell Tissue Res. 2012;347(3):701–711.

17. Petrenko Y, Syková E, Kubinová Š. The therapeutic potential of three‑dimensional multipotent mesenchymal stromal cell spheroids. Stem Cell Res Ther. 2017;8(1):94.

18. Cesarz Z, Tamama K. Spheroid culture of mesenchymal stem cells. Stem Cells Int. 2016;2016:9176357.

19. Grayson WL, Zhao F, Bunnell B, Ma T. Hypoxia enhances proliferation and tissue formation of human mesenchymal stem cells. Biochem Biophys Res Commun. 2007;358(3):948–953.

20. Yamada Y, Okano T, Orita K, Makino T, Shima F, Nakamura H. 3D‑cultured small size adipose‑derived stem cell spheroids promote bone regeneration in the critical‑sized bone defect rat model. Biochem Biophys Res Commun. 2022;603:57–62.

21. Shima F, Makino T. Fabrication of spheroids with dome‑shaped endothelial tube networks by an adhesive culture system. Adv Biosyst. 2020;4(10):2000120.

22. NIPPON SHOKUBAI CO., LTD. 3D Cell Culture Vessel: MicoCell. Product information page. Available from: https://www.shokubai.co.jp/en/products/detail/micocell/ (accessed 27 September 2025).

23. Ministry of Health, Labour and Welfare (Japan). Act on the Safety of Regenerative Medicine (ASRM). Available from: https://www.pmda.go.jp/english/review-services/reviews/0003.html (accessed 27 September 2025).

24. Japan Registry of Clinical Trials (jRCT). Study jRCTb050200097. Available from: https://jrct.mhlw.go.jp/en-latest-detail/jRCTb050200097 (accessed 27 September 2025).

25. National Cancer Institute. Common Terminology Criteria for Adverse Events (CTCAE) v5.0. 2017. Available from: https://dctd.cancer.gov/research/ctep-trials/for-sites/adverse-events/ctcae-v5-5x7.pdf (accessed 27 September 2025).

26. International Council for Harmonisation (ICH). Clinical Safety Data Management: Definitions and Standards for Expedited Reporting (E2A). 1994. Available from: https://database.ich.org/sites/default/files/E2A_Guideline.pdf (accessed 27 September 2025).
